# High-frequency random DNA insertions upon co-delivery of CRISPR-Cas9 ribonucleoprotein and selectable marker plasmid in rice

**DOI:** 10.1038/s41598-019-55681-y

**Published:** 2019-12-27

**Authors:** Raviraj Banakar, Alan L. Eggenberger, Keunsub Lee, David A. Wright, Karthik Murugan, Scott Zarecor, Carolyn J. Lawrence-Dill, Dipali G. Sashital, Kan Wang

**Affiliations:** 1 Department of Agronomy, Iowa State University, Ames, IA USA; 2 Crop Bioengineering Center, Iowa State University, Ames, IA USA; 3 Plant Transformation Facility, Iowa State University, Ames, IA USA; 4 Roy J Carver Department of Biochemistry, Biophysics and Molecular Biology, Iowa State University, Ames, IA USA; 5 Department of Genetics, Development and Cell Biology, Iowa State University, Ames, IA USA; 6 Present Address: Department of Plant and Microbial Biology, University of Minnesota, Saint Paul, MN USA

**Keywords:** Biotechnology, Plant sciences

## Abstract

An important advantage of delivering CRISPR reagents into cells as a ribonucleoprotein (RNP) complex is the ability to edit genes without reagents being integrated into the genome. Transient presence of RNP molecules in cells can reduce undesirable off-target effects. One method for RNP delivery into plant cells is the use of a biolistic gun. To facilitate selection of transformed cells during RNP delivery, a plasmid carrying a selectable marker gene can be co-delivered with the RNP to enrich for transformed/edited cells. In this work, we compare targeted mutagenesis in rice using three different delivery platforms: biolistic RNP/DNA co-delivery; biolistic DNA delivery; and *Agrobacterium*-mediated delivery. All three platforms were successful in generating desired mutations at the target sites. However, we observed a high frequency (over 14%) of random plasmid or chromosomal DNA fragment insertion at the target sites in transgenic events generated from both biolistic delivery platforms. In contrast, integration of random DNA fragments was not observed in transgenic events generated from the *Agrobacterium*-mediated method. These data reveal important insights that must be considered when selecting the method for genome-editing reagent delivery in plants, and emphasize the importance of employing appropriate molecular screening methods to detect unintended alterations following genome engineering.

## Introduction

The CRISPR-Cas (clustered regularly interspaced short palindromic repeat/CRISPR-associated) system has been a method of choice for precise genome editing in many organisms, including plants. Successful CRISPR-mediated editing experiments have been reported in many plant species including rice, corn, wheat, and soybean^1–3^. The CRISPR-Cas system generates precise double-strand breaks (DSBs) at DNA target sites. To survive the DSBs, the DNA has to be repaired to maintain cellular homeostasis^4–7^. DSBs are mainly repaired by either homology directed recombination (HDR)^8,9^, which creates precise editing by copying sequence information from a donor template^10,11^, or homologous end joining (NHEJ). NHEJ repair is predominant in somatic plant cells that are often the target cells used in plant transformation^8–11^. Unlike precise HDR, NHEJ often introduces short insertions or deletions (indels) at the DSBs, generating loss-of-function mutations by creating frame shifts leading to premature stop codons.

CRISPR reagents are generally introduced into plants cells by biolistic- and *Agrobacterium*-mediated plant transformation^12^. In the case of *Agrobacterium*-mediated transformation, CRISPR reagents are introduced as DNA molecules by placing Cas9 and gRNA expression cassettes within the transfer DNA (T-DNA)^13^. Once *Agrobacterium* delivers the T-DNA into the plant cell, the expression of the CRISPR reagents are enabled, resulting in genome editing^14^. In general, the *Agrobacterium*-mediated transformation method has been widely used in plant genome editing due to the simplicity of the method as well as the ability of *Agrobacterium* to transfer large fragments of DNA containing numerous genes^15,16^. Most importantly, *Agrobacterium* has the propensity to generate single or low copy number integration events with defined termini; thus, it is less disruptive to the plant genome^17^.

Despite the advantages of *Agrobacterium*-mediated transformation and its widespread use to transform a large variety of organisms, a number of problems remain. One issue is that T-DNA integration into the host genome is random^17^. Thus, T-DNA can integrate into transcriptionally silent regions, or other regions of the genome that result in transgene silencing or expression instability. This lack of transgene expression predictability and stability can confound molecular analyses and increase costs of generating edited plants for research or commercial purposes. Random T-DNA integration can also result in disruption of host genes or genomic regions important for organismal growth, development, or agronomic characteristics. It has also been reported that T-DNA integration in plant genomes is not always precise, but that vector backbone DNA can be transferred with the T-DNA^18,19^. In addition, T-DNA insertions can cause large-scale genome re-arrangement in plants^20^.

In contrast to *Agrobacterium*-mediated transformation, biolistics utilize physical force to introduce DNA molecules into plant cells^21^. Therefore, it is not limited by complex interactions with the host plant required for *Agrobacterium*-mediated transformation. In addition, the biolistic method has the ability to deliver oligonucleotides, mRNA and proteins into plant cells^11,22,23^. However, because of the physical force used for biolistic transformation, DNA shearing is prominent, thus generating a range of DNA fragments available for DNA repair.

For the purpose of genome editing, it is often desirable for the CRISPR reagents to be present in cells in a transient fashion. Expression of CRISPR-Cas from a DNA cassette means that the DNA can integrate into the plant genome and cause continued genome editing and off-target effects in subsequent generations. Delivery of CRISPR reagents as ribonucleoproteins (RNPs), which have a limited half-life, has been used to avoid this problem^11,24^. However, using biolistics for RNP delivery in plants has a low transformation frequency due to the small number of cells receiving microprojectiles in the bombardment. As a consequence, large-scale screening is needed to identify edited plants^11,23,24^. To circumvent this, selectable marker genes such as antibiotic- or herbicide-resistance genes can be co-delivered with RNP to facilitate the enrichment of transformed cells^11^. In this case, it is expected that gene editing occurs transiently once the RNP complex enters a cell. Co-delivered plasmid DNAs carrying the selectable marker gene will randomly integrate into the genome, allowing the transformed and edited cells to be selected on culture media containing selective agents. If desired, the selectable marker transgene can be segregated from the edited locus through segregation in subsequent generations.

Although DNA molecules delivered using both the *Agrobacterium* and biolistic methods are randomly integrated into the genome, some can be inserted into DSBs generated by the CRISPR reagents^25,26^. Therefore, it is important to examine the outcomes of CRISPR reagents delivery when using different transformation platforms. In this work, three different transformation platforms, biolistic-mediated RNP/DNA co-delivery, biolistic-mediated DNA delivery, and *Agrobacterium*-mediated DNA delivery, were used to deliver CRISPR reagents for targeted mutagenesis of a rice phytoene desaturase gene (*OsPDS*). All three transformation methods successfully generated indel mutations at the target sites. Intriguingly, we observed that random DNA fragments, originated mostly from plasmid DNA but some from chromosomal DNAs, were inserted at the CRISPR target sites when biolistic delivery platforms were used for transformation. In contrast *Agrobacterium*-mediated transformation did not result in integration of random DNA at the target site.

## Results

### Biolistic co-delivery of CRISPR-Cas9 RNP and selectable marker plasmid DNA leads to integration of DNA fragments at target site

**Figure 1:**
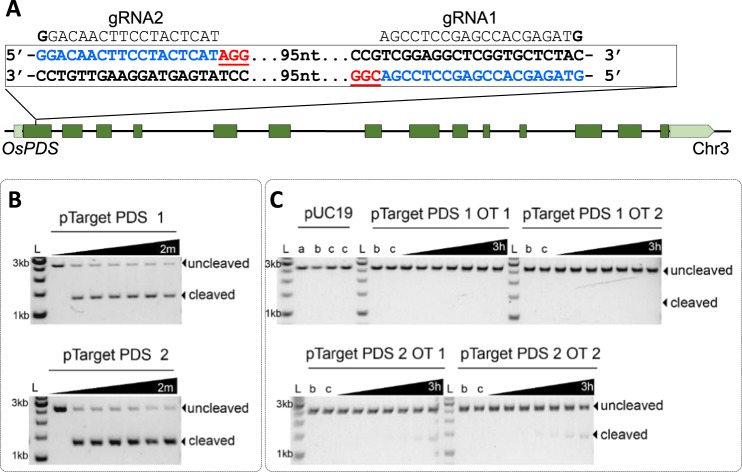
Guide RNA design and *in vitro* cleavage analysis. (**A**) Rice phytoene desaturase gene (*OsPDS*) on chromosome 3. Dark green boxes represent exons, light green box and box arrow represents 5′ and 3′ untranslated regions, respectively. Two gRNA target sequences (in exon 1) are in blue and PAM sequences are in red. **(B)** *In vitro* cleavage assay for two target sequences PDS1 and PDS2. **(C)** *In vitro* cleavage assay for top two off-target sequences with highest homologies for PDS1 and PDS2. 2 m, 2 minutes; 30 m, 30 minutes; 3 h, 3 hours. a = target plasmid alone, b = target plasmid + gRNA 1 or 2, c = target plasmid + SpCas9, L = 1 kb DNA ladder.

We reasoned that the use of a visual phenotypic alteration in plants would be useful to track the progress of our genome editing experiments. Therefore, we chose rice phytoene desaturase (*OsPDS1-* Os03g0184000) as a target. The *OsPDS1* is a single copy gene with 14 exons located on rice chromosome 3 (Fig. 1A). This gene is involved in a biochemical process of converting phytoene to carotene in plants^27^. Knocking out of the *PDS* gene renders leaves sensitive to photo-bleaching. In this work, we have targeted the first coding exon (referred to here as exon 1) of *OsPDS1* so that editing would result in a gene knock out and the appearance of an albino phenotype.

Two guide RNAs (gRNAs) were designed using the CRISPR Genome Analysis Tool^28^ (CGAT, http://cbc.gdcb.iastate.edu/cgat/, Supplementary Table S1). Twenty-base-pair (20-bp) gRNA1 targeted the antisense strand of the *PDS*, while 18-bp gRNA2 targeted the sense strand at sites 95-bp apart (Fig. 1A). These two target sequences along with their PAM sequence (Supplementary Table S2) were cloned into pUC19 for *in vitro* cleavage efficiency analysis^29^. Figure 1B shows that both guides were cleaved efficiently by commercial SpCas9 (ALT-R S.P. CAS9, Integrated DNA Technology, USA). The observed cleavage rate is 35 ± 3 min^−1^ for *PDS* target-1 and 31 ± 3 min^−1^ for *PDS* target-2. These results suggested that there was no marked difference between the two guides for their cleavage efficiency *in vitro*.

We also selected the top two potential off-target sites for each gRNA predicted by CGAT (Supplementary Tables S3 & S4). These off-target sites with highest sequence homologies to each gRNA target (Supplementary Tables S3 & S4) were subjected to *in vitro* cleavage assay using ALT-R S.P. CAS9 (Fig. 1C). Compared to on-target cleavage (Fig. 1B), these off-target sequences appeared to have very low cleavage efficiencies, suggesting that both guide RNAs would provide specific on-target editing.

**Figure 2:**
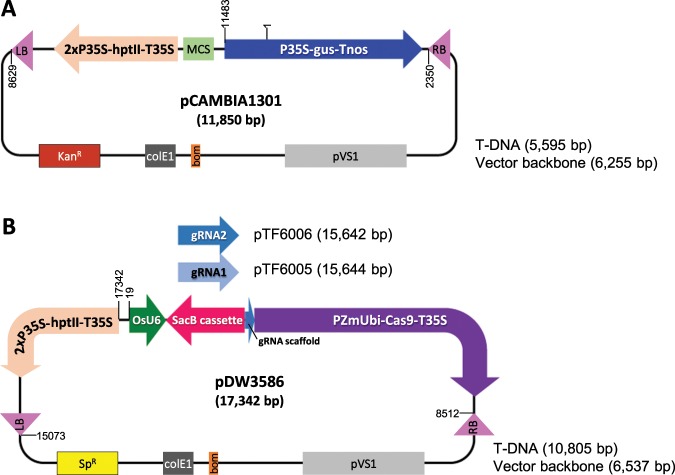
Schematic illustration of plasmid constructs used for rice transformation. (**A**) pCAMBIA1301 (Roberts *et al*. 1996) used for the co-delivery of CRISPR RNA complex and plasmid DNA. (**B**) pDW3586 (this work) used for the construction of pTF6005 and pTF6005, which carries PDS gRNA1 and gRNA2, respectively. RB, right border; LB, left border; 2xP35S-hptII-T35S, hygromycin resistance gene cassette; P35S-gus-Tnos, beta-glucuronidase (*GUS*) report gene cassette; PZmUbi-Cas9-T35S, maize ubiquitine promoter driving Cas9 expression cassette; OsU6, Oryza sativa U6 small RNA promoter; SacB cassette, *B*. *subtilis* counter-selectable marker gene for facilitating the cloning of gRNAs; MCS, multiple cloning site; pVS1, replication origin from Pseudomonas aeruginosa; bom, *E*. *coli* origin of transfer; colE1, replication origin plasmid ColE1; Kan^R^, kanamycin resistance gene; Sp^R^, spectinomycin resistance gene; numbers on the plasmids refer to sequence coordinates.

**Figure 3:**
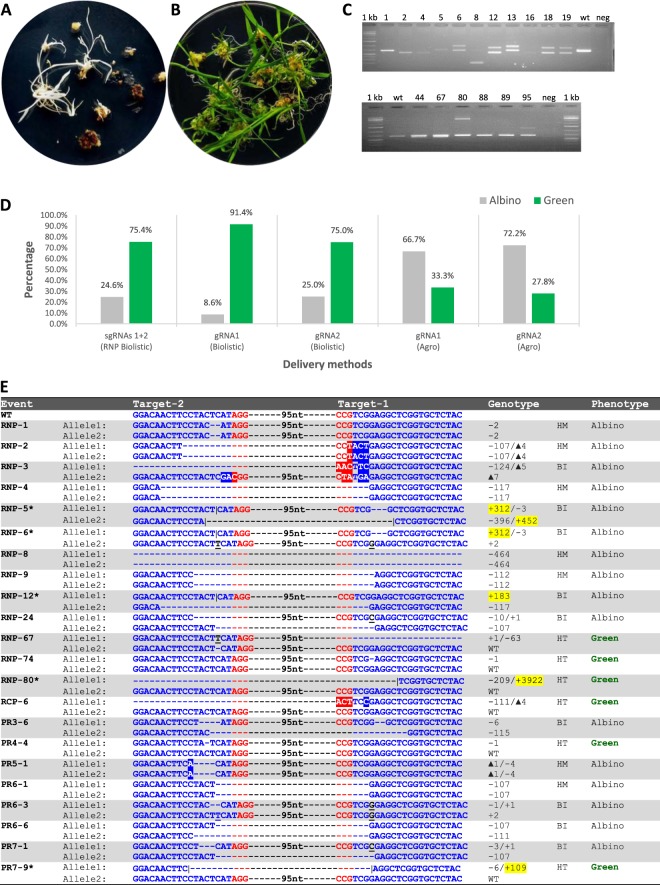
CRISPR-RNP delivery in rice. (**A**) Hygromycin resistant albino rice regenerants. **(B)** Hygromycin resistant green rice regenerants. **(C)** Both top and bottom gels represent agarose gel electrophoresis showing PDS amplicon, numbers represent independent transformation lines; 1 kb, molecular weight marker; wt, wild type; neg, water control. **(D)** Percentages of albino and green regenerants produced by three different transformation platforms. **(E)** Mutations in T0 transgenic lines. Blue letters, target sequences in *PDS* exon 1; Red letters, PAM sequences; White letters in blue or red boxes, substitution mutations; Black letter with underscore, insertion mutations; Event marked with star, event with random DNA inserted at the target sites; black vertical lines, positions where random DNA inserted; yellow highlighted numbers, sizes of random DNA fragments; WT, wild type; HM, homozygous; BI, biallelic; HT, heterozygous.

Having established the *in vitro* cleavage ability of the two designed guides, we then set out to test the *in vivo* rice genome editing ability of CRISPR-RNP molecules. We delivered pre-assembled CRISPR-RNP complex (gRNA1-SpCas9 + gRNA2-SpCas9) into rice tissue using the PDS-1000/HE BIOLISTIC PARTICLE DELIVERY SYSTEM. The rice target tissue was mature seed-derived embryos of Japonica rice (Nipponbare) that were cultured on callus induction medium for 7 days. To facilitate effective selection in rice tissue culture and transformation, we co-bombarded plasmid DNA pCAMBIA1301 (Fig. 2A) with the RNP reagents. pCAMBIA1301^30^ (GenBank accession number AF234297.1) carries the antibiotic hygromycin B resistance cassette that is commonly used for rice transformation. The PDS mutant phenotype was observed in the second round of selection as colorless calli that were resistant to hygromycin and proliferated in the culture. Both hygromycin resistant albino (Fig. 3A) and green plants (Fig. 3B) were recovered.

A total of 65 hygromycin resistant events were recovered from bombarded embryos. Out of the 65 transgenic events, 16 (24.6%) were albino (none were fertile) and 49 (75.4%) were green (Table 1, Fig. 3D). Of the 49 green events only 19 events were fertile. To genotype these events, we first selected pairs of primers (Supplementary Table S5) from the genomic region surrounding gRNA1 and gRNA2 target sites and used these primers to generate DNA amplions using PCR. As shown in Fig. 3C, different sizes of amplicon products were visible for some events on the agarose gel. Therefore, co-delivery of two sgRNAs targeting two sites that are 95-bp apart and on different DNA strands generated a variety of mutations that allowed preliminary screening of edited plants using a simple electrophoresis technique.

**Table 1:** Summary of on-target mutation of T0 rice transgenic plants generated using three different CRISPR reagent delivery platforms*.

Delivery method	Phenotype	# Hyg res lines	# Fertile	# Analyzed	# PDS mutated	Mutation type^Ɨ^				# Random DNA insertion event
						Homozygous	Biallelic	Heterozygous	Mosaic	
RNP Cas9 + sgRNAs(1 + 2) (Biolistic)	Albino	16	0	16	16	44% (7)	56% (9)	0% (0)	0% (0)	3
	Green	49	19	19	6	0% (0)	0% (0)	100% (6)	0% (0)	2
pTF6005 (gRNA1) (Biolistic)	Albino	3	0	3	3	67% (2)	33% (1)	0% (0)	0% (0)	1
	Green	32	10	10	3	0% (0)	67% (2)	33% (1)	0% (0)	0
pTF6006 (gRNA2) (Biolistic)	Albino	5	0	5	5	40% (2)	60% (3)	0% (0)	0% (0)	1
	Green	15	3	3	2	0% (0)	0% (0)	100% (3)	0% (0)	1
pTF6005 (gRNA1) (Agro)	Albino	4	0	4	4	25% (1)	25% (1)	0% (0)	50% (2)	0
	Green	2	1	1	1	0% (0)	100% (1)	0% (0)	0% (0)	0
pTF6006 (gRNA2) (Agro)	Albino	13	0	13	13	15% (2)	85% (11)	0% (0)	0% (0)	0
	Green	5	1	1	1	0% (0)	100% (1)	0% (0)	0% (0)	0

^*^Numbers in parentheses represent number of plants analyzed.

^Ɨ^Homozygous, two identical mutant sequences; Biallelic, two different mutant sequences; Heterozygous, wild type sequence and one mutant sequence; Mosaic, multiple mutations sequences.

To characterize the targeted mutation lines, PCR fragments amplified from all albino plants and fertile green plants (35 events in total) were cloned and subjected to Sanger sequencing. Eight independent clones of each PCR fragment were typically sequenced. As shown in Tables 1, 62.9% (22 out of 35 events) of the transgenic events generated by RNP-DNA co-delivery carried mutations (Fig. 3E). As expected, all 16 albino events had either homozygous or bi-allelic mutations. On the other hand, only 6 out of the 19 fertile green events (31.6%) had mutation and all of them were heterozygous mutants (Fig. 3E, Table 1). Five out of 22 mutant events (22.7%, Fig. 3E) had simple indel mutations (RNP-1, −74, PR4-4, PR5-1, and PR6-3), while the remaining events had either large deletions or a combination of large deletions and insertions.

Interestingly, out of the 35 events analyzed for mutation, five events (Fig. 3E, events marked with stars) had unexpected insertion of DNA fragments from the selectable marker plasmid or chromosomal DNA at the target site with an insertion frequency of 14.3% (5 out of 35) of the total number of transgenic events analyzed and 22.7% (5 out of 22) of the mutant events (Table 1, Fig. 3E). In particular, three of these insertion events were albino events and two were fertile green events.

### Biolistic delivery of CRISPR-Cas9 reagents as plasmid DNA leads to insertion of DNA fragments at target site

We next delivered CRISPR-Cas9 reagents as DNA molecules to determine if that would also lead to insertion of plasmid DNA fragments at the target site. DNA sequences of two gRNAs (gRNA1 and gRNA2) used in RNP experiments were cloned separately into a vector pDW3586 (Fig. 2B). By replacing the SacB cassette with gRNA1 and gRNA2 sequences in vector pDW3586, we created the constructs pTF6005 and pTF6006 (Fig. 2B).

**Figure 4:**
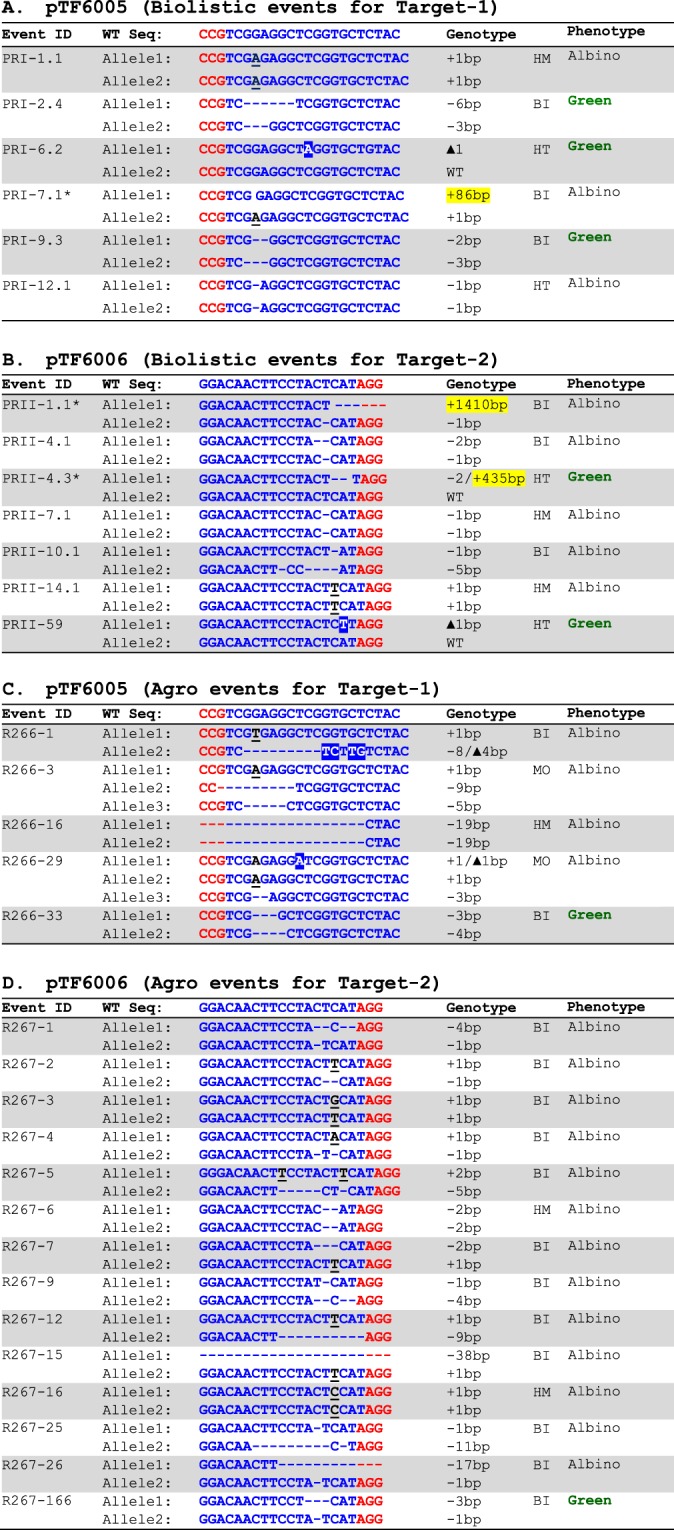
CRISPR-DNA delivery using either biolistic- or *Agrobacterium*-mediated transformation methods. (**A**) Mutations from biolistic delivery of pTF6005 (PDS target-1). **(B)** Mutations from biolistic delivery of pTF6006 (PDS target-2). **(C)** Mutations from *Agrobacterium* delivery of pTF6005 (PDS target-1). **(D)** Mutations from *Agrobacterium* delivery of pTF6006 (PDS target-2). Blue letters, target sequences in *PDS* exon 1; Red letters, PAM sequences; White letters in blue or red boxes, substitution mutations; Black letter with underscore, insertion mutations; Event marked with star, event with random DNA inserted at the target sites; black vertical lines, positions where random DNA inserted; yellow highlighted numbers, sizes of random DNA fragments; WT, wild type; HM, homozygous; BI, biallelic; HT, heterozygous; MO, mosaic.

These plasmids were introduced independently into rice as described in the Materials and Methods. In the case of the gRNA1 construct pTF6005, we generated a total of 35 independent transgenic events with 3 albino events and 32 green events (Fig. 3D, Table 1). Of the three albino events, two albino events were homozygous mutants and one event carried a bi-allelic mutation (Fig. 4A). One albino event (PRI-7.1) had an insertion of an 86-bp DNA fragment at the target site (Fig. 4A). Out of the 32 green events, only three of the 10 fertile events were determined to be mutants. Of the three green mutant events, one event had a heterozygous mutation, but the two other events carried bi-allelic mutations (Fig. 4A). DNA sequence analysis revealed that both events have in-frame nucleotide deletions. Event PRI-2.4 had bi-allelic deletions of 3- and 6-bp, respectively, on each allele; while event PRI-9.3 had 2- and 3-bp deletions, respectively, on each allele (Fig. 4A).

Similarly, with the gRNA2 construct pTF6006 we generated a total of 20 independent transgenic events of which 5 and 15 events were albino and green events, respectively (Fig. 3D, Table 1). Again, all five albino events were either homozygous (2 events) or bi-allelic (3 events) mutants. Of the three green fertile events analyzed, two events had heterozygous mutations at the target site (Fig. 4B). One albino (PRII-1.1) and one fertile green (PRII-4.3) event had insertion of 1438-bp and 437-bp DNA fragments, respectively, at the target site (Table 1, Fig. 4B).

To verify whether the random DNA fragment insertions at target sites were dependent on creation of DSBs by Cas9, we performed control experiments in which rice tissue were bombarded with either pDW3586 (Fig. 2B) or pCAMBIA1301 (Fig. 2A) vector DNA only. pDW3586 is a SpCas9 expressing plasmid without guide RNA and pCAMBIA1301 has no CRISPR reagents. A total of 10 and 18 independent transgenic events were generated for pDW3586 and pCAMBIA1301, respectively. As expected, all of the events were green, but only 3 out of 10 pDW3586 events and 5 out of 18 pCAMBIA1301 evets were fertile. Genotypic analysis of the target sites revealed no indels or insertions in any of the events. These results demonstrate that insertion of random DNA fragments at target sites indeed depends on creation of DSBs by CRISPR-Cas9.

### *Agrobacterium*-mediated delivery of CRISPR reagents does not result in insertion of random DNA fragments at target site

To determine whether integration of random DNA fragments at target sites is unique to biolistic-mediated delivery of CRISPR reagents, we carried out *Agrobacterium*-mediated transformation of rice using *Agrobacterium* strain EHA101 harboring the constructs pTF6005 and pTF6006 (Fig. 2B), which were used in biolistic delivery of CRISPR reagents as DNA molecules. A total of 5 and 14 Agro events were analyzed for pTF6005 and pTF6006, respectively (Table 1). As shown in Fig. 3D and Table 1, in the case of *Agrobacterium*-mediated delivery, a higher percentage of albino vs green plants were recovered compared to that from the biolistic DNA delivery experiments. With the gRNA1 construct (pTF6005), 66.7% of Agro events were albinos compared to 8.6% of albino biolistic events. Similarly, 72.2% albino Agro events were identified compared to 25.0% of albino biolistic events from the gRNA2 construct (pTF6006). Genotyping analyses were carried out on all albino and fertile green plants. As can be seen from Table 1 and Fig. 4C,D, all Agro events analyzed for the two constructs had mutations at the targeted sites. For the four pTF6005 albino Agro events, two events were either homozygous or bi-allelic mutants, the remaining two events carried more than two types of mutant sequences, suggesting they were mosaic mutations (Fig. 4C). One green pTF6005 event (R266-33) had a 3-bp in-frame deletion on one allele (Fig. 4C). For pTF6006 Agro transformation, all 13 albino plants carried mutations, with the majority (85%) being bi-allelic mutants (Fig. 4D). One green event (R267-166) carried a 3-bp in-frame deletion on one allele (Fig. 4D). None of the Agro events had insertion of either plasmid or chromosome DNA fragments at the target sites (Table 1, Fig. 4C,D), nor was a T-DNA insertion observed. Although statistically not significant due to the small sample size (5/56 for biolistic methods vs. 0/19 for *Agrobacterium*-mediated method, Table 1; ***z = ***1.74, ***P*** = 0.08, two proportions ***z*** test^31^), these results demonstrate that biolistic methods can result in more frequent insertion of random plasmid DNA fragments at target sites than does the *Agrobacterium*-mediated transformation.

### Characterization of inserted DNA fragments reveals simple and complex sequence arrangements

**Figure 5:**
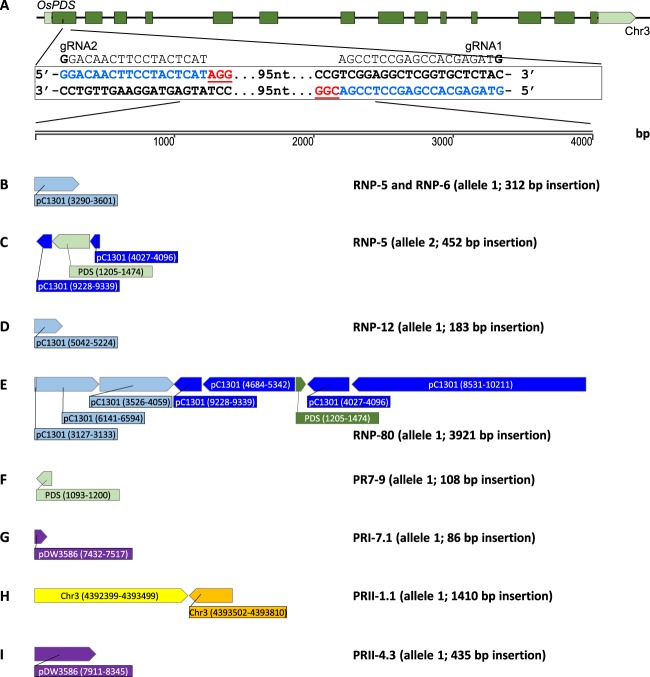
Schematic illustration and composition of the random DNA fragments in PDS target sites of eight mutant lines. (**A**) Rice phytoene desaturase gene (*OsPDS*) on chromosome 3. Dark green boxes represent exons, light green box and box arrow represents 5′ and 3′ untranslated regions, respectively. Two gRNA target sequences (in exon 1) are in blue and PAM sequences are in red. bp, base pair. (**B**–**I**) DNA fragments inserted in five RNP events (RNP-5, RNP-6, RNP-12, RNP-80 and PR7-9), and three biolistic-DNA events (PRI-7.1, PRII-1.1 and PRII-4.3).

The sequences of five random DNA insertion events from the co-delivery of RNP molecules and pCAMBIA1301 plasmid DNA (event RNP-5, RNP-6, RNP-12, RNP-80 and PR7-9, Fig. 3E) and three events from the biolistic DNA delivery of CRISPR reagents (event PRI-7.1, PRII-1.1 and PRII-4.3, Fig. 4A,B) were further analyzed. The details of these random DNA inserts are shown in Fig. 5 and Supplementary files 1 to 8.

RNP event RNP-5 (Fig. 3E) was a bi-allelic insertion event. A 312-bp DNA fragment from the pCAMBIA1301 vector backbone (coordinates 3290–3601, Fig. 2B) was inserted at target-2 of allele 1 (Figs. 3E and 5B). A 3-bp deletion was detected at target-1 (2-bp downstream of PAM) of the same allele. At allele 2, a deletion of 396-bp was detected, which started from 3-bp upstream of the PAM in target-2 and extending 291-bp beyond the cleavage site of target-1. A 452-bp fragment was inserted into the sites, which included 112-bp from the T-DNA of pCAMBIA1301 (coordinates 9228–9339), 270-bp from the PDS1 gene (coordinates 1205–1474), and 70-bp from the vector backbone of pCAMBIA1301 (coordinates 4027–4096) (Figs. 2A and 5C).

Event RNP-6 was similar to event RNP-5 for allele 1 (Figs. 3E and 5B), carrying a 312-bp fragment of the pCAMBIA1301 vector backbone (coordinates 3290–3601) at target-2 and a 3-bp deletion at target-1. On allele 2, this event had two single base-pair insertions, a T at target-2 and a G at target-1. In both cases the insertion occurred 3-bp upstream of the PAM sequence (Fig. 3E). The fact that both RNP-5 and −6 had the identical insertions and deletions on allele 1 is intriguing. There is no homology between the sites surrounding the inserted fragment and the insertion region. One possible explanation is that they were derived from a single heterozygous mutant callus event at an early stage of transformation, but developed into two different mutant events further in the regeneration process due to different mutations on allele 2. However, because these two mutants were produced by RNP delivery in which CRISPR reagents were short lived, it is unlikely due to the continuous mutagenesis by RNP molecules. Further DNA insertion analysis of the selectable marker gene insertion location could reveal whether they were clonal for the transgene.

Event RNP-12 was also a bi-allelic mutant with an insertion on one of the alleles (Figs. 3E and 5D). On allele 1, a 183-bp fragment of the pCAMBIA1301 vector backbone (coordinates 5042–5224, Fig. 2B) was inserted 5-bp upstream of the target-2 PAM site. Target-1 on the same allele remained un-edited. On allele 2, a 117-bp fragment was deleted between 13-bp upstream of target-2 PAM site and 3-bp upstream of target-1 PAM site.

Event RNP-80 was a heterozygous insertion event in which there were multiple insertions in allele 1 but allele 2 remained un-changed (Fig. 3E). This event had a deletion of a 209-bp sequence which extended 83-bp upstream of the target-2 sequence and 8-bp downstream of the target-1 PAM sequence. The deletion was replaced by eight random DNA fragments with a total size of 3921-bp, seven were from pCAMBIA1301 and one fragment from the PDS coding sequence (Fig. 5E). Although this event had complex insertions, it was green and fertile due to the un-mutated PDS gene on allele 2.

Another green insertion RNP event PR7-9 (Fig. 3E) was also a heterozygous mutant. It seems that the 107-bp PDS fragment, which was cut out by the two gRNAs, was re-joined into the cut site after an inversion. Both gDNA ends, i.e., the 5′ of gRNA2 cut site and the 3′ of the gRNA1 cut site, had short indels: a 5-bp deletion (CTACT) + 1-bp insertion (T) at the gRNA2 cut site and 1-bp deletion (G) + 1-bp insertion (A) at the gRNA 2 cut site. These gDNA ends were re-joined with the inverted 107-bp PDS fragment, presumably by the NHEJ pathway (Fig. 5F).

Biolistic DNA delivery of the gRNA1 construct pTF6005 (Fig. 2B) generated three albino plants, one of which had DNA insertion at the target site (Table 1, Fig. 4A). This event PRI-7.1 had an 86-bp insertion from T-DNA region (coordinates 7432–7517) at target-1 on allele 1 (Figs. 4A & 5G). On allele 2, a single base (A) insertion took place 3-bp away from the PAM sequence (Fig. 3A).

Two insertion events resulted from the biolistic DNA delivery of the gRNA2 construct pTF6006 (Fig. 2B), one was albino and one was green (Fig. 4B). For the albino event PRII-1.1, the target-2 acquired a 1410-bp insertion consisting of two pieces of rice chromosome 3, one was 1101-bp (coordinates 4392399–4393499) and the second one was 309-bp (coordinates 4392399–4393499) (Fig. 5H). On allele 2 there was a deletion of a single base-pair (T) 3-bp upstream of the PAM sequence (Fig. 4B).

For the green and fertile event PRII-4.3, its allele 1 had a 2-bp deletion at the target-2 (1-bp upstream of PAM) and a 435-bp insertion of the T-DNA region (coordinates 7911–8345) of pTF6006 (Figs. 2B, 4B & 5I). Allele 2 of this event was not modified.

### Mutations are inherited to the next generation

Seven events representing three different reagent delivery platforms mentioned in Table 2 were fertile, hence, these events were forwarded to the next generation for inheritance analysis. As can be seen in Table 2, in the T1 generation, both green and albino plants could be obtained from all these lines with different albino to green ratios. Selected seedlings were genotyped and the mutation patterns of these events were inherited in their T1 progenies. For example, heterozygous mutant event RNP-67 produced both green and albino seedlings in the T1 generation. Green plants inherited the parental genotype, but albino plants carried the same mutations on both alleles. Biolistic event PRI-9.3 was a bi-allelic mutant with a 2-bp and 3-bp deletion on each allele. The T0 plant was green because the 3-bp deletion did not produce a frame shift and PDS function was not affected. However, in the progeny, one of the six plants was albino and had a 2-bp deletion on both alleles. A similar observation was made on *Agrobacterium* event R266–33, which was a green plant with a 4-bp deletion on one allele in T0 generation. In the progeny, one of four seedlings was albino carrying the 4-bp deletion on both alleles, while the other three T1 plants had no mutations (Table 2).

**Table 2:** T0 genotyping, DNA copy number estimation, and T1 inheritance analysis of selected mutant lines.

Event ID	T0^a^		T1^b^ (Phenotype/Genotype)					
	Phenotype/Genotype	DNA Copy #	T1-1	T1-2	T1-3	T1-4	T1-5	T1-6
**RNP events**								
RNP-67	Green/(0, +1/−63)	3	Albino/(+1/−63, +1/−63)	Albino/(+1/−63, +1/−63)	Green/(0, 0)	Green/(0, +1/−63)		
RNP-74	Green/(0, −1)	3	Albino/(−1, −1)	Albino/(−1, −1)	Albino/(−1, −1)			
RNP-80	Green/(0, −209/+3922)	1	Green/(0, −209/+3922)	Green/(0, −209/+3922)	Green/(0, −209/+3922)	Green/(0, −209/+3922)		
**Biolistic events**								
PRI-9.3	Green/(−2, −3)	2	Albino/(−2, −2)	Green/(−2, −3)	Green/(−2, −3)	Green/(−3, −3)	Green/(−3, −3)	Green/(−3, −3)
PRII-4.3	Green/(0, −2/+435)	2	Green/(0, −2/+435)	Green/(0, −2/+435)	Green/(0, 0)	Green/(0, 0)		
**Agro events**								
R266-33	Green/(−4, 0)	1	Green/(0, 0)	Green/(0, 0)	Green/(0, 0)	Albino/(−4, −4)		
R267-166	Green/(−1, −3)	2	Green/(0, −1)	Green/(−1, −3)				
**Control event**								
R289-9	Green/(N/A)	2	Green/(N/A)	Green/(N/A)	Green/(N/A)			

^a^T0, mutant genotypes in T0 plants; Numbers in the parenthesis indicate indel sizes.

^b^T1, mutant genotypes in T1 plants; T1-1, −2 to −6, sibling T1 plants. Numbers in the parenthesis indicate indel sizes.

N/A, not analyzed.

To determine whether any editing experiments resulted in off-target editing, we also examined off-target mutations in seven mutant lines representing three CRISPR reagent delivery platforms (Table 2). In two of the off-target sites tested no mutations were observed (Supplementary Tables S5 & S6). This is consistent with the low cleavage rates observed for these off-targets in *in vitro* assays (Fig. 1B). Furthermore, the same seven events were analyzed for transgene copy number using *hptII* gene fragment as proxy. As shown in Table 2, all events had a low DNA copy number (between 1–3 copies) integrated in the genome.

## Discussion

Delivery of CRISPR reagents to cells as ribonucleoprotein (RNP) is a common practice in mammalian research^32^, and is also becoming one of the methods of choice for plant research^33^. The delivery of Cas9/gRNA as an RNP complex allows the transient presence of the reagents to achieve gene editing in the cell, avoids CRISPR reagent integration in the genome, and reduces potential off-target activities. One of the challenges in delivering RNPs into plant cells is the need to enrich the transformed/edited cell/tissue during the process. One practice involves co-bombarding a plasmid DNA carrying an antibiotic- or herbicide- resistance gene with the CRISPR RNP complex to assist in the selection of transformed/edited plant tissue^11^. In this work, we report the observation of high frequency random DNA fragment insertion at the CRISPR target site when using the RNP/DNA co-delivery strategy for gene mutagenesis in rice. Over 1/5 of the mutated events had insertions at the targeted sites. This phenomenon was also observed with similar frequency in mutant events that were generated using biolistic DNA delivery methods. Most of the random DNA fragments were derived from the plasmids carrying the hygromycin-resistant marker gene, while some insertions were rice chromosomal DNA. Large insertions were composed of multiple smaller fragments.

It is known that biolistic-mediated DNA delivery can lead to insertion of plasmid DNA, often fragmented, into random genome locations in plants^34,35^. However, this unintended DNA insertion is underreported in the literature and when reported, the detailed data were often not supplied in the publications. For example, papers that described work in human cell lines using Zinc Finger Nuclease reported template plasmid integration at both targeted and off-target sites^36,37^. Similar observations were made in *C*. *elegans*^38^ and fish^39^ using the CRISPR-Cas9 system. Most recently, unintended on-site template DNA insertion was reported as key findings in work with CRISPR edited mice^40^ and cattle^41^.

The unintended DNA integration observed in this study might be due in part to procedural differences. The RNP/DNA co-delivery described in this work used chemically synthesized guide RNA that were generated by a commercial company Integrated DNA Technologies Inc. (IDT, Coralville, IA, USA). A recent study has reported that synthetic gRNAs were more effective than *in vitro* transcribed gRNAs in achieving genome editing^42^. One other procedural difference is that a transfection reagent *TRANS*IT-2020 (Mirus Bio LLC, Madison, WI), instead of spermidine, was used for both biolistic platforms. *TRANS*IT-2020 is a commercial lipid-polymer mixture that has been widely used for plasmid DNA transfection into mammalian cells (https://www.mirusbio.com/tech-resources/faqs/transit-2020-faqs). Recently, it was used successfully for biolistically delivering RNP/DNA into plant cells^11^. Because the majority of random DNA fragments were originated from the co-bombarded plasmid DNA, future experiments should be conducted to determine whether reducing the amount of plasmid DNA used in the co-delivery would be helpful to decrease the on-site random DNA insertion events. For delivery of RNP without a selectable marker gene, fragmented chromosomal DNA generated by the biolistic transformation can still be inserted into target sites, but this is expected to occur at a much lower frequency.

This work compared three different but commonly used transformation platforms for the delivery of CRISPR reagents in plants. All three methodologies were successful in generating intended mutations at the target sites, though frequencies in generating albino phenotypes varied. In the RNP/DNA co-delivery, RNPs containing two gRNAs targeting the same *PDS* gene were mixed before being introduced into the cells. This mixed gRNA delivery produced mutation events with large deletions across the both gRNA targeting sites that are 95 nucleotides apart. Random DNA insertions were observed from both biolistic delivery platforms with similar frequency (14%). On the other hand, no random DNA insertion event was detected from the mutants generated by *Agrobacterium*-mediated transformation. Nor did we detect any T-DNA insertion events in these mutant lines. Our group has recently demonstrated that CRISPR-Cas9-mediated targeted T-DNA integration could be possible in rice^26^. Between 4–5% of transgenic rice callus lines had targeted T-DNA insertion with precise sequences at the T-DNA right-border. Small sample sizes obtained in this work (5 events with gRNA1 and 14 events with gRNA2) might explain the failure of observing targeted T-DNA integration.

In plant research, unintended on-site DNA integration events are likely to be missed through traditional genome editing analysis. Because undesired genome arrangement can be readily segregated out in progenies of seed plants through hybridization, detailed investigation of abnormity is mostly neglected due to resource limitations. However, our work shows that the unintended on-site random DNA insertion frequency can be high when employing the CRISPR-Cas9 system. Therefore, this outcome should not be overlooked.

Our work highlights the importance of molecular screening and the strategy for screening both on- and off-target sites. When the focus and biased assumption is on intended edits, genome rearrangements and unintended insertions can go undetected. For example, the widely used CRISPR analysis tools such as CRISPResso, CRISPR-RGEN, TIDE and ICE analysis are designed for short read analysis. Furthermore, PCR conditions that are designed to amplify a short region including the target site may fail to amplify an unintended insertion, leading to an incorrect characterization of the editing event. There are indeed reports that PCR failed to detect multiple integration events^25,40,41^, thus PCR reagents and extension times should be selected to amplify much larger than expected fragments. For edited lines that are intended for commercialization, it will be necessary to perform long read sequencing or whole genome sequencing to determine exact nature of edited events. Successful identification of unintended insertion and genome rearrangements are important information for further improvement of the CRISPR-mediated genome editing for plants.

## Materials and Methods

### Guide RNA design and *in vitro* cleavage assay

The rice *PDS* gene (Os03g0184000, NCBI reference sequence NC_029258.1) encoding phytoene desaturase, which functions in the carotenoid biosynthesis pathway, was chosen as the target gene for CRISPR-mediated editing. To verify the sequence, primer pairs (PDS1F- TGAATATAATTTTAGGAG and PDS1R- CAATGCTAAGACCACGATGTGA, Supplementary Table S5) were designed to amplify a 525-bp fragment (985–1509 bp within the reference sequence) using Primer3 version 4.1.0 software^43^ (https://www.prime3software.com/). The gene was amplified from the rice japonica cultivar Nipponbare (obtained from the Agriculture Research Service, United States Department of Agriculture, Stuttgart, AR, USA), cloned into pJET1.2 (ThermoFisher Scientific, MA, USA) and multiple plasmid clones were sequenced.

Two target sites, GTAGAGCACCGAGCCTCCGACGG (23-bp, targeted by crRNA1 or gRNA1**)** and GGACAACTTCCTACTCATAGG (21 bp, targeted by crRNA2 or gRNA2) (Fig. 1A, Supplementary Table S1) in the anti-sense and sense strand of the first exon of PDS, respectively, were selected using the CRISPR Genome Analysis Tool^28^ (CGAT, http://cbc.gdcb.iastate.edu/cgat/) developed by Iowa State University. Each target sequence was unique within the rice genome without any off-target sites with equal to or fewer than two mismatches. To test the *in vitro* cleavage efficiency of these guides, complementary oligos (Supplementary Table S2) containing the target sequences were obtained from Integrated DNA Technologies (IDT, Coralville, IA, USA), and cloned into pUC19. The resulting target plasmids (pTarget) were subjected to Sanger sequencing to verify the sequences. Target plasmids were linearized using *Bsa*I-HF digestion prior to the cleavage assay.

*In vitro* cleavage assays were performed as described^29^, using crRNA, tracrRNA, and SpCas9 obtained from IDT. RNPs were assembled according to the manufacturer’s instructions, using 1:1.5 molar ration of SpCas9 to annealed crRNA and trRNA. Cleavage reactions were initiated by mixing pTargets (150 ng) with RNP (100 nM: 150 nM SpCas9: tracr-crRNA final concentration) and incubating at 37 °C. Aliquots were withdrawn from the reaction at each time point and electrophoresed on an agarose gel. Bands were visualized and quantified with image analysis software ImageQuant TL v8.1.0.0 https://www.gelifesciences.com/en/us/shop/protein-analysis/molecular-imaging-for-proteins/imaging-software/imagequant-tl-8-1-p-00110 (GE Healthcare Life Sciences, PA, USA). The intensities of bands in the cleaved and uncleaved fractions were measured, and the fraction cleaved was calculated as cleaved fraction/(cleaved fraction + uncleaved fraction). Observed cleavage rates were obtained by fitting the fraction cleaved in to a one-phase association rate equation using GraphPad Prism 6 v7.05 (https://www.graphpad.com/scientific-software/prism/).

### Rice transformation vector construction

Rice transformation vectors pTF6005 and pTF6006 (Fig. 2B) for biolistic- and *Agrobacterium*-mediated transformation were created by cloning crRNA1 (TCGGAGGCTCGGTGCTCTA) and crRNA2 (GGACAACTTCCTACTCAT) into vector pDW3586 using *BsaI* (Supplementary file 9). Vector pDW3586 was based on a construct pUbi-Cas9 system described previously^44^.

### CRISPR-RNP complex formation and gold coating

Custom synthesized crRNA1 (2 nmol), crRNA2 (2 nmol), tracrRNA (5 nmol), and SpCas9 (67 µmol) were purchased from IDT, and stored at −20 °C until use. Briefly, on the day of CRISPR-RNP delivery to plant cells, crRNA and tracrRNA were dissolved in 20 µL of nuclease free-IDTE buffer (1 × -TE buffer, pH 7.5) to a concentration of 100 µM of each. To form the gRNA complex, equimolar concentration crRNAs (100 µM of crRNA1 and crRNA2 each) and tracrRNA (200 µM) were mixed in 1.5 mL centrifuge tubes and placed in a 95 °C heat block for 5 min. After 5 min the tubes were centrifuged at 15,871 xg (13,000 rpm) for 5 s and placed at RT (22 °C) for 10 min. In order to form the RNP complex, 67 µM (10 µL) of SpCas9 was added along with 2 µL of 1 × -PBS buffer (pH 7.4) to this tube. The solution was mixed by pipetting up and down, and the tube was incubated at RT for 10 min. This RNP complex was used for biolistic delivery into rice embryos.

To coat the RNP complex onto gold particles, 7 µL of RNP complex were transferred to a tube containing 0.75 mg of gold in 25 µL sdH_2_O, prewarmed to RT and sonicated. For selection of transformed rice cells, 500 ng of pCAMBIA1301 plasmid was added to the gold and RNP complex. Plasmid DNA was prepared using QIAprep Spin Miniprep Kit, Midi prep or Maxi Prep (Qiagen Inc-USA, Germantown, MD), by following the manufacturer’s protocol.

Gold, RNP and plasmid DNA were mixed by pipetting up and down and the tube was placed on ice. To this mixture, 2 µL of water-soluble cationic lipid TransIT-2020 (Mirus Bio LLC, Madison, WI) was added and the solution was mixed thoroughly by pipetting and incubated on ice for 10 min^11^. The tube was then centrifuged at 845 xg (3000 rpm) for 30 s, the supernatant was discarded, and the pellet was dissolved in 40 µL of sdH_2_O. The mixture was sonicated for 10 s to homogenize the gold-RNP-DNA mixture, and four 10 µl aliquots were placed on four macrocarrier discs in the laminar flow hood. Macrocarriers were allowed to dry in the hood, which normally took 15–20 min.

### Biolistic-mediated rice transformation

Procedures for embryo isolation, osmotic treatment, post-osmotic recovery, selection, regeneration and plant care were performed as previously described^45–48^ with modification^49^. Briefly, seeds were germinated on MS medium with 2 mg/L 2,4-D for 6 days at 27 °C in the dark. On the 7^th^ day embryos were plated on osmoticum medium^49^ for 4 h before bombardment. RNP/DNA complex or plasmid DNA alone were coated onto gold and bombarded using the PDS-1000/HE BIOLISTIC PARTICLE DELIVERY SYSTEM, with 900 psi rupture discs and a 6 cm target distance. Embryos were kept on the same osmoticum media for 16 h (27 °C, dark) post-biolistic delivery. The embryos were then transferred to resting media^49^ for 24 h (27 °C, dark), followed by two rounds of selection (15 days each) that were performed on MS selection media containing 50 mg/L hygromycin. Surviving calli were transferred to MS regeneration medium^49^ with 6-benzylaminopurine (BAP, 3 mg/L), and 1-naphthaleneacetic acid (NAA, 0.25 mg/L). Fully established albino and green shoots were obtained within 15 days in regeneration media and were transferred to ½ MS media without hormones for rooting. Rooted albino seedlings were collected and stored at –80 °C, whereas, the green seedlings were transferred to soil. Plant growth care and maintenance were performed as described^49^. Leaf tissue was collected from 1-month-old green plants and stored at –80 °C.

### *Agrobacterium*-mediated rice transformation

Secondary calli derived from mature rice seeds of cultivar Nipponbare were used as explants for *Agrobacterium*-mediated transformation as previously described^50^. The transformation experiments were performed in Iowa State University Plant Transformation Facility.

### Genotyping

Genomic DNA was isolated from leaves of transgenic rice plants as described previously^51^. PCR screening of genomic DNAs was performed with high-fidelity PrimeStar GXL DNA Polymerase (Takara Bio, CA, USA), using 50 ng of DNA and following the manufacturer’s protocol. PDS1 forward primer (TGAATATAATTTTAGGAG), and PDS1 reverse primer (TCACATCGTGGTCTTAGCATTG) were used to amplify a fragment of the PDS gene surrounding the gRNA1 and gRNA2 target sites (Table S1). PCR products were gel-purified using a QIAquick gel extraction kit (Qiagen Inc-USA, Germantown, MD), and cloned into vector pJET1.2 using a CloneJET PCR Cloning Kit (ThermoFisher Scientific, MA, USA) as per manufacturers instruction. Sequencing of clones was performed at the DNA Facility at Iowa State University (Ames, IA, USA). Sequence analysis was performed by analyzing chromatograms using SnapGene 5.0 Viewer (https://www.snapgene.com/snapgene-viewer/), and performing alignments with Clustal Omega (https://www.ebi.ac.uk/Tools/msa/clustalo/) and NCBI BLAST (https://blast.ncbi.nlm.nih.gov/Blast.cgi?PAGE_TYPE=BlastSearch).

### Transgene copy number analysis

Transgene copy numbers were estimated as described previously^26^. Briefly, a single copy reference gene *OsUBC*^52^ (Os02g42314) and *hptII* gene fragments were PCR amplified and cloned into pJET1.2 (ThermoFisher Scientific, MA, USA), resulting in a control vector pKL1026^26^. After sequence verification by sequencing, pKL1026 DNA was serially diluted and quantitative PCR (qPCR) was performed on a Mx3005p qPCR system (Agilent Technologies, Inc., Germany) using a Qiagen RT^2^ SYBR Green master mix to generate standard curves. About 5 ng of genomic DNA was used for each 25 µl qPCR reaction. Estimated copy numbers of *OsUBC* and *hptII* were used to calculate transgene copy numbers. Because the reference gene *OsUBC* has two alleles in the rice genome, a 1:2 ratio of *hptII* copy number to that of *OsUBC* was interpreted as a single copy transgene event.

### Off-target mutation analysis

For both crRNA1 and crRNA2, a number of possible off-target sites were identified with CGAT^28^ (Supplementary Tables S3 and S4). These sites differed from the target sites by more than 2-bp. Genomic DNA flanking two of the top three ranked off-target sites for each gRNA (Supplementary Tables S3 and S4) were amplified with primers designed with Primer3 software^43^. Analysis of the off-target sites was performed as described for the target sites using primer pairs listed in Supplementary Table S5.

### Analysis of mutation inheritance

Seeds were harvested from fertile transgenic plants at physiological maturity. Seeds were placed in an envelope and air dried at 37 °C for 3 days and then stored at room temperature until use. Seeds of both wild type and transgenic plants were germinated on ½ MS media without the selection agent. Seedlings were scored two weeks after germination as albino or green. Multiple individual seedlings were used for DNA extraction, PCR amplification and sequencing as previously described.

### Statistical analysis

A two proportions ***z*** test^31^ was used to compare the frequencies of random DNA insertions at the target sites by biolistic (RNP/DNA co-delivery and DNA delivery) and *Agrobacterium*-mediated methods.

## Supplementary information

Supplementary information
