# Supplementary material for: High-frequency random DNA insertions upon co-delivery of CRISPR-Cas9 ribonucleoprotein and selectable marker plasmid in rice
Source: Sci Rep. 2019 Dec 27;9:19902. doi: 10.1038/s41598-019-55681-y (PMC6934568; doi:10.1038/s41598-019-55681-y)
Supplement: Supplementary file 1 — Supplementary information [file 41598_2019_55681_MOESM1_ESM.pdf]

Supplementary Materials

High-frequency random DNA insertions upon co-delivery of CRISPR-Cas9 ribonucleoprotein and selectable marker plasmid in rice

Raviraj Banakar[1,2 #¶], Alan L. Eggenberger[1,2], Keunsub Lee[1,2], David A. Wright[2,3], Karthik Murugan[2,4], Scott Zarecor[2,5], Carolyn J. Lawrence-Dill[1,2,5], Dipali G. Sashital[2,4], Kan Wang[1,2 *]

[1] Department of Agronomy, Iowa State University, Ames, IA, USA

[2] Crop Bioengineering Center, Iowa State University, Ames, IA, USA.

[3] Plant Transformation Facility, Iowa State University, Ames, IA, USA.

[4] Roy J Carver Department of Biochemistry, Biophysics and Molecular Biology, Iowa State University, Ames, IA, USA.

[5] Department of Genetics, Development and Cell Biology, Iowa State University, Ames, IA, USA.

* Corresponding author:

Kan Wang

Email: kanwang@iastate.edu

# Current address: Department of Plant and Microbial Biology, University of Minnesota, Saint Paul, MN, USA

Table S1. Oligonucleotides used for cloning guides to target rice PDS1 gene.

Oligo name	Sequence
gRNA1-Oligo1	tgttGTAGAGCACCGAGCCTCCGA
gRNA1-Oligo2	aaacTCGGAGGCTCGGTGCTCTAC
gRNA2-Oligo1	tgttGGACAACTTCCTACTCAT
gRNA2-Oligo2	aaacATGAGTAGGAAGTTGTCC

Table S2: Oligonucleotides used for in vitro cleavage efficiency analysis. Oligos containing BamHI and EcoRI restriction sites used for cloning DNA targets and off-targets into plasmid (Bold = PAM, RED = mismatch).

Oligonucleotides	Sequence
gRNA1 target oligo1	AATTCGTAGAGCACCGAGCCTCCGA**CGG**G
gRNA1 target oligo2	CCCGTCGGAGGCTCGGTGCTCTACGAATT
gRNA1 off-target 1 oligo1	AATTCGGCGAGCACCGAGCAGCCGA**TGG**G
gRNA1 off-target 1 oligo2	GATCCCCATCGGCTGCTCGGTGCTCGCCG
gRNA1 off-target 2 oligo1	AATTCGCCGAACGCCGtGCCTCCGA**TGG**G
gRNA1 off-target 2 oligo2	GATCCCCATCGGAGGCACGGCGTTCGGCG
gRNA2 target oligo1	AATTCGGGACAACTTCCTACTCAT**AGG**G
gRNA2 target oligo2	GATCCCCTATGAGTAGGAAGTTGTCCCG
gRNA2 off-target 1 oligo1	AATTCGGAACACCTTCCTAGTCAT**TGG**G
gRNA2 off-target 1 oligo2	GATCCCCAATGACTAGGAAGGTGTTCCG
gRNA2 off-target 2 oligo1	AATTCGGAACAACTTCCTCCTCAA**GGG**G
gRNA2 off-target 2 oligo2	GATCCCCCTTGAGGAGGAAGTTGTTCCG

Table S3: Off-target sites prediction by CGAT (gRNA1). Potential off-targets are compared with target gRNA1 (GTAGAGCACCGAGCCTCCGACGG). Sequence base pair difference between gRNA1 and off-target sites are highlighted in red. Two potential off-target sites highlighted in yellow are used for in vitro cleavage assay and rice off-target analysis.

Off-target	Match Sequence	Gene	Position	Strand	5' Identity	3' Identity	Repeats	GC
**Off-1**	GGCGAGCACCGAGCAGCCGATGG	OS08T0432300-00	785	+	60%	86%	2	0.74
**Off-2**	GTCGCCCACCGATCATCCGATGG	OS05T0440300-01	3558	-	40%	86%	3	0.65
**Off-3**	GCCGAACGCCGTGCCTCCGATGG	OS04T0573300-00<br>OS04T0573200-01<br>OS04T0573200-02	463<br>250<br>170	+<br>+<br>+	40%	86%	2	0.74
**Off-4**	GGTGGACACCGCGCCACCGAGGG	OS09T0363800-00	575	-	20%	86%	3	0.78
**Off-5**	GACCTCCACCGAGCCGGCGATGG	OS05T0382000-01	3224	-	0%	86%	2	0.74
**Off-6**	GCCTCCCACTGAGGCTCCGACGG	OS07T0661900-01	1919	+	0%	86%	3	0.74
**Off-7**	GAGCTCCACCGAGCCACCAATGG	OS06T0619132-00<br>OS06T0619600-00<br>OS06T0619650-00	843<br>1527<br>1501	-<br>+<br>+	0%	86%	2	0.65
**Off-8**	GACGAACACCGAGTCGACGAGGG	OS01T0770400-01	3174	+	40%	79%	3	0.65
**Off-9**	GAGCAGCAGCAAGCCGCCGATGG	OS02T0203200-01<br>OS02T0203300-00	893<br>849	+<br>+	40%	79%	2	0.70
**Off-10**	GAAGCTCACCGAGCCTGCTCAGG	OS08T0440300-01<br>OS08T0440300-03<br>OS08T0440300-04	950<br>840<br>74	-<br>-<br>-	40%	79%	2	0.65
**Off-11**	GACGGCGACGGGGCCTCCGAAGG	OS01T0618200-01	553	+	20%	79%	4	0.78
**Off-12**	GACCAACGCCGAGCTGCCGATGG	OS02T0725900-01	693	-	20%	79%	2	0.70
**Off-13**	GACTCGCAGCGAGCCAGCGATGG	OS07T0256866-00	77	+	20%	79%	2	0.70
**Off-14**	GAACGTCGCCAAGCCACCGAAGG	OS11T0210600-01	2066	-	20%	79%	2	0.65
**Off-15**	GACATGGACCGCGCCTCCGTCGG	OS05T0237400-01<br>OS05T0237501-00	2758<br>379	-<br>-	20%	79%	2	0.74
**Off-16**	GAGGGACAGCGAGCCAGCGAGGG	OS05T0134000-03<br>OS05T0134000-01	3744<br>3705	-<br>-	20%	79%	3	0.74
**Off-17**	GAGCGACGTCGAGCCGCCGACGG	OS03T0254400-00	927	-	0%	79%	2	0.78
**Off-18**	GAGAGCCACAGAGCCGCCGCCGG	OS09T0526800-02<br>OS09T0526800-01	2868<br>2680	+<br>+	0%	79%	2	0.78
**Off-19**	GACCTTCACCGACCCGCCCACGG	OS09T0459450-01<br>OS09T0459500-00	365<br>301	+<br>+	0%	79%	3	0.74

Table S4: Off-target sites prediction by CGAT (gRNA2). Potential off-targets are compared with target gRNA2 (GACAACTTCCTACTCATAGG). Sequence base pair difference between gRNA2 and off-target sites are highlighted in red. Two potential off-target sites highlighted in yellow are used for in vitro cleavage assay and rice off-target analysis.

Off-target	Match Sequence	Gene	Position	Strand	5' Identity	3' Identity	repeats	GC
**Off-1**	GAACACCTTCCTAGTCATTGG	OS06T0304600-01<br>OS06T0304600-02	5656<br>5618	-<br>-	60%	92%	2	0.48
**Off-2**	GCAAATCTTCGTACTCATAGG	OS03T0784800-02<br>OS03T0784800-01	3880<br>3880	+<br>+	40%	92%	3	0.43
**Off-3**	GAACAACTTCCTCCTCAAGGG	OS08T0515600-01<br>OS08T0515700-01	1932<br>1780	-<br>-	80%	83%	3	0.52
**Off-4**	GAACAACTACCTCCTCATTGG	OS08T0179000-01<br>OS08T0179100-00<br>OS08T0179150-01	5672<br>630<br>705	+<br>+<br>+	80%	83%	2	0.48
**Off-5**	GCAGAACCTCCTCCTCATTGG	OS11T0696200-01	2580	-	60%	83%	2	0.57
**Off-6**	GAACACCATCCTAGTCATTGG	OS01T0304200-00	2405	+	60%	83%	2	0.48
**Off-7**	GAAGGACTGCCTACTCAAAGG	OS04T0566100-00	2427	+	40%	83%	3	0.52
**Off-8**	GAACTCATTGCTACTCATGGG	OS08T0234200-01	2119	-	40%	83%	3	0.48
**Off-9**	GCATATCTTCCTATTGATTGG	OS03T0291800-01<br>OS03T0291800-02<br>OS03T0291800-03	2455<br>1948<br>585	-<br>-<br>-	40%	83%	2	0.38
**Off-10**	GCCCTTCTTCATCCTCATCGG	OS03T0185400-01	1028	+	20%	83%	3	0.57
**Off-11**	GAGTATGGTCCTACTCATTGG	OS01T0869900-01	1874	+	20%	83%	2	0.48
**Off-12**	GAAGGGCTTCCTCCTCAAAGG	OS03T0279900-01	1294	-	20%	83%	3	0.57
**Off-13**	GCCCTGCTTCCTAATTATTGG	OS06T0681300-02	2098	+	20%	83%	3	0.48
**Off-14**	GAAGCTCGTCCTATTCATTGG	OS03T0210050-00	51	-	20%	83%	2	0.48
**Off-15**	GAAGCTCTTCCTTCTCCTTGG	OS05T0141500-02<br>OS05T0141500-01	2480<br>2434	+<br>+	20%	83%	2	0.52
**Off-16**	GCCTAGCTTCATCCTCATCGG	OS08T0125200-01<br>OS08T0125250-00	1607<br>1483	-<br>-	20%	83%	2	0.57
**Off-17**	GCAGGTCTTCCTTCTCCTTGG	OS03T0233800-01<br>OS03T0233800-02<br>OS03T0233750-00	3241<br>3025<br>753	-<br>-<br>-	20%	83%	2	0.57
**Off-18**	GCCTTGCTTACTCCTCATTGG	OS02T0453600-01	497	-	0%	83%	2	0.52
**Off-19**	GCGATTCTTCATACTCACCGG	OS10T0525200-01	3654	-	0%	83%	2	0.52

Table S5: Primer pairs used for on target and off target analysis in rice.

Primer name	Forward primer	Reverse primer
**On target**		
PDS1	TGAATATAATTTTAGGAG	CAATGCTAAGACCACGATGTGA
PJET1.2	CGACTCACTATAGGGAGAGCGGC	AAGAACATCGATTTTCCATGGCAG
**Off targets**		
OsPDS1-gRNA1-Off-1	AGGGCAGTCTCAACCCATAA	AAGAAAAGGGGCAGAGCATC
OsPDS1-gRNA1-Off-3	TGATCCACCTGATCATGATTGT	CCAAAAGCTGAAGAAAAGCTG
OsPDS1-gRNA2-Off-1	AGTGGCTGCTCACCCAGTC	CATGACAGAGGAAGGCCAAA
OsPDS1-gRNA2-Off-3	TTCAGAACGAAAAGCTCGTG	ATCGCAGCAAACCACCTAGT

Table S6: Off target analysis in T0 generation plants derived from *Agrobacterium*, biolistics, and RNP delivery. Base pairs shown in red color represent bases differing between gRNA and off target site. WT, wild type; G, Green

**gRNA1**			
**Event**	**Sequence**	**Genotype**	**Phenotype**
**Off-1**			
WT	GGCGAGCACCGAGCAGCCGATGG		
R-266-3-3	GGCGAGCACCGAGCAGCCGATGG	WT	G
PRI-9.3-1	GGCGAGCACCGAGCAGCCGATGG	WT	G
RNP-67-3	GGCGAGCACCGAGCAGCCGATGG	WT	G
RNP-74-2	GGCGAGCACCGAGCAGCCGATGG	WT	G
RNP-80-2	GGCGAGCACCGAGCAGCCGATGG	WT	G
R-267-16-6	GGCGAGCACCGAGCAGCCGATGG	WT	G
PRII-4.6-1	GGCGAGCACCGAGCAGCCGATGG	WT	G
**Off-3**			
WT	GCCGAACGCCGTGCCTCCGATGG		G
R-266-3-3	GCCGAACGCCGTGCCTCCGATGG	WT	G
PRI-9.3-1	GCCGAACGCCGTGCCTCCGATGG	WT	G
RNP-67-3	GCCGAACGCCGTGCCTCCGATGG	WT	G
RNP-74-2	GCCGAACGCCGTGCCTCCGATGG	WT	G
RNP-80-2	GCCGAACGCCGTGCCTCCGATGG	WT	G
R-267-16-6	GCCGAACGCCGTGCCTCCGATGG	WT	G
PRII-4.6-1	GCCGAACGCCGTGCCTCCGATGG	WT	G
**gRNA2**			
**Event**	**Sequence**	**Genotype**	**Phenotype**
**Off-1**			
WT	GAACACCTTCCTAGTCATTGG		
R-267-16-6	GAACACCTTCCTAGTCATTGG	WT	G
PRII-4.6-1	GAACACCTTCCTAGTCATTGG	WT	G
RNP-67-3	GAACACCTTCCTAGTCATTGG	WT	G
RNP-74-2	GAACACCTTCCTAGTCATTGG	WT	G
RNP-80-2	GAACACCTTCCTAGTCATTGG	WT	G
R-266-3-3	GAACACCTTCCTAGTCATTGG	WT	G
PRI-9.3-1	GAACACCTTCCTAGTCATTGG	WT	G
**Off-3**			
WT	GAACAACTTCCTCCTCAAGGG		
R-267-16-6	GAACAACTTCCTCCTCAAGGG	WT	G
PRII-4.6-1	GAACAACTTCCTCCTCAAGGG	WT	G
RNP-67-3	GAACAACTTCCTCCTCAAGGG	WT	G
RNP-74-2	GAACAACTTCCTCCTCAAGGG	WT	G
RNP-80-2	GAACAACTTCCTCCTCAAGGG	WT	G
R-266-3-3	GAACAACTTCCTCCTCAAGGG	WT	G
PRI-9.3-1	GAACAACTTCCTCCTCAAGGG	WT	G

Figure Legends for Supplementary files 1 to 9:

All files are constructed using SnapGene 5.0 Viewer (https://www.snapgene.com/snapgene-viewer/)

PDS1: Rice phytoene desaturase gene

pCAMBIA1301: plasmid used for the co-delivery of CRISPR RNA complex and plasmid DNA (see Figure 2)

gRNA1 and gRNA2: Oligonucleotides of PDS1 gene (Table S1)

PDS1F and 1R, PDS2F and 2R: primer pairs for on-target analysis (Table S5)

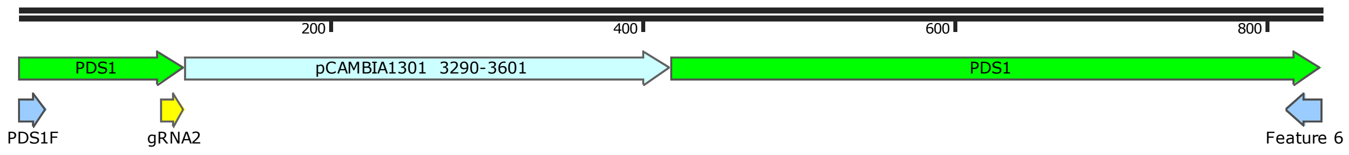

RNP-5 allele 1 and RNP-6 allele 1
834 bp

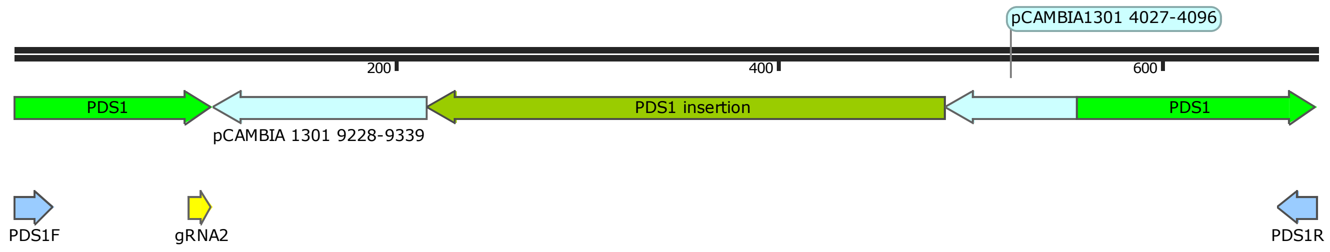

SF2_RNP-5 (allele 2)
680 bp

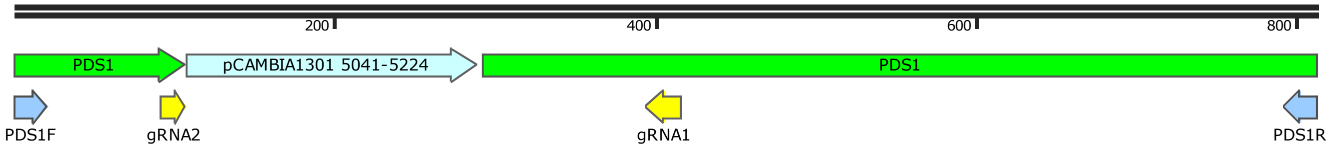

RNP-12 (allele 1)
811 bp

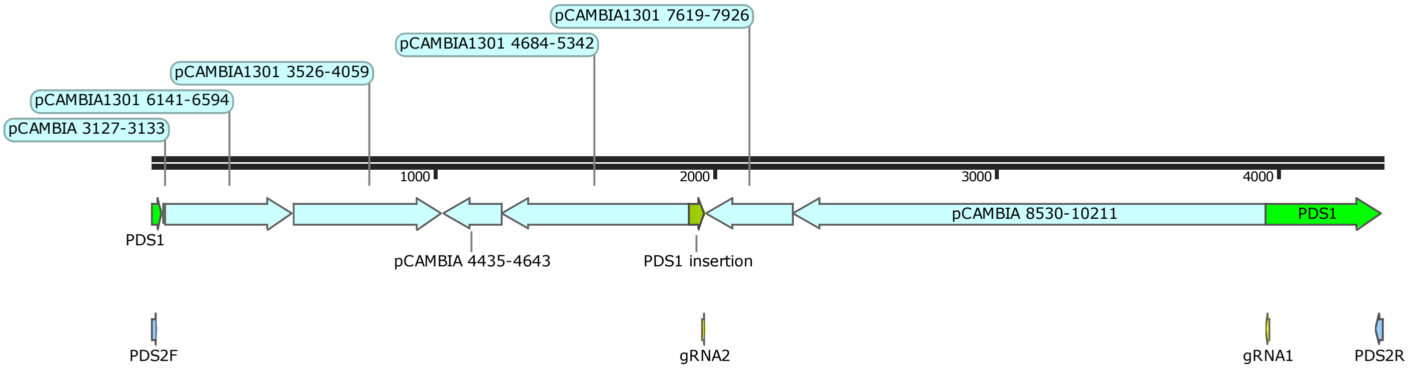

RNP-80 (allele 1)
4370 bp

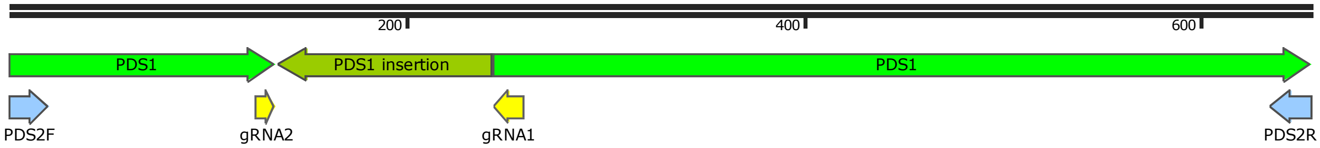

PR7-9 (allele 1)
654 bp

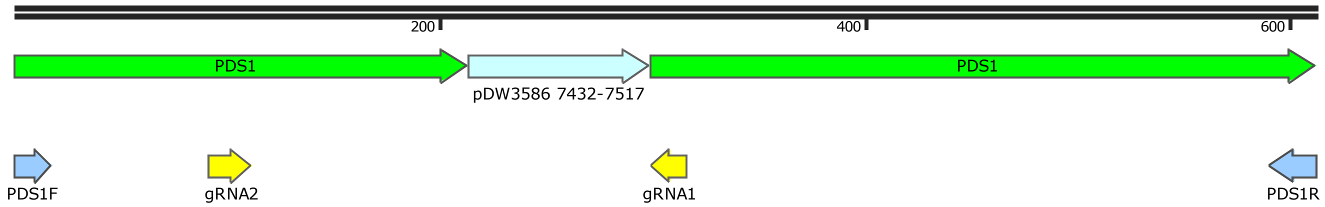

PRI-7.1 (allele 1)
611 bp

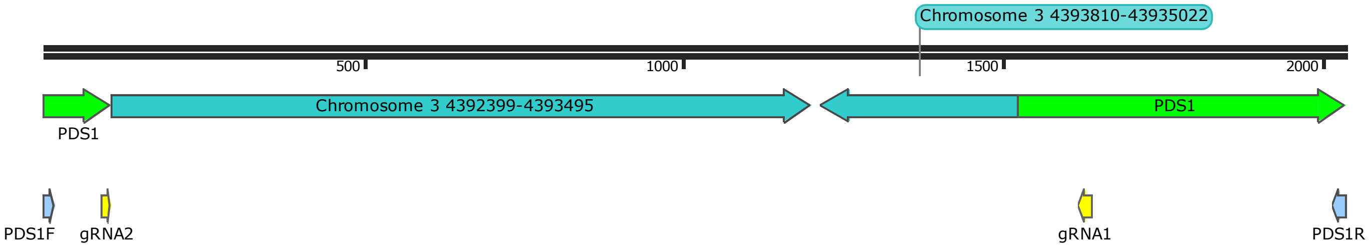

PRII-1.1 (allele 1)
2033 bp

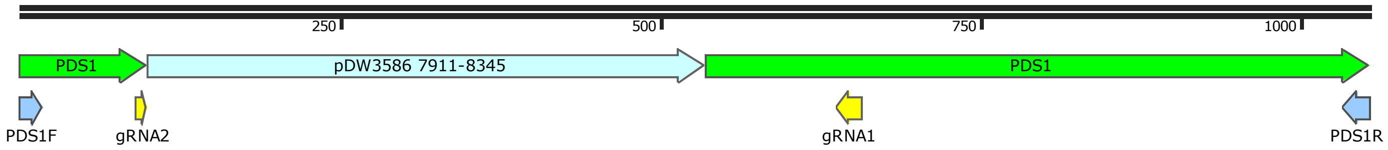

PRII-4.3 (allele 1)
1052 bp

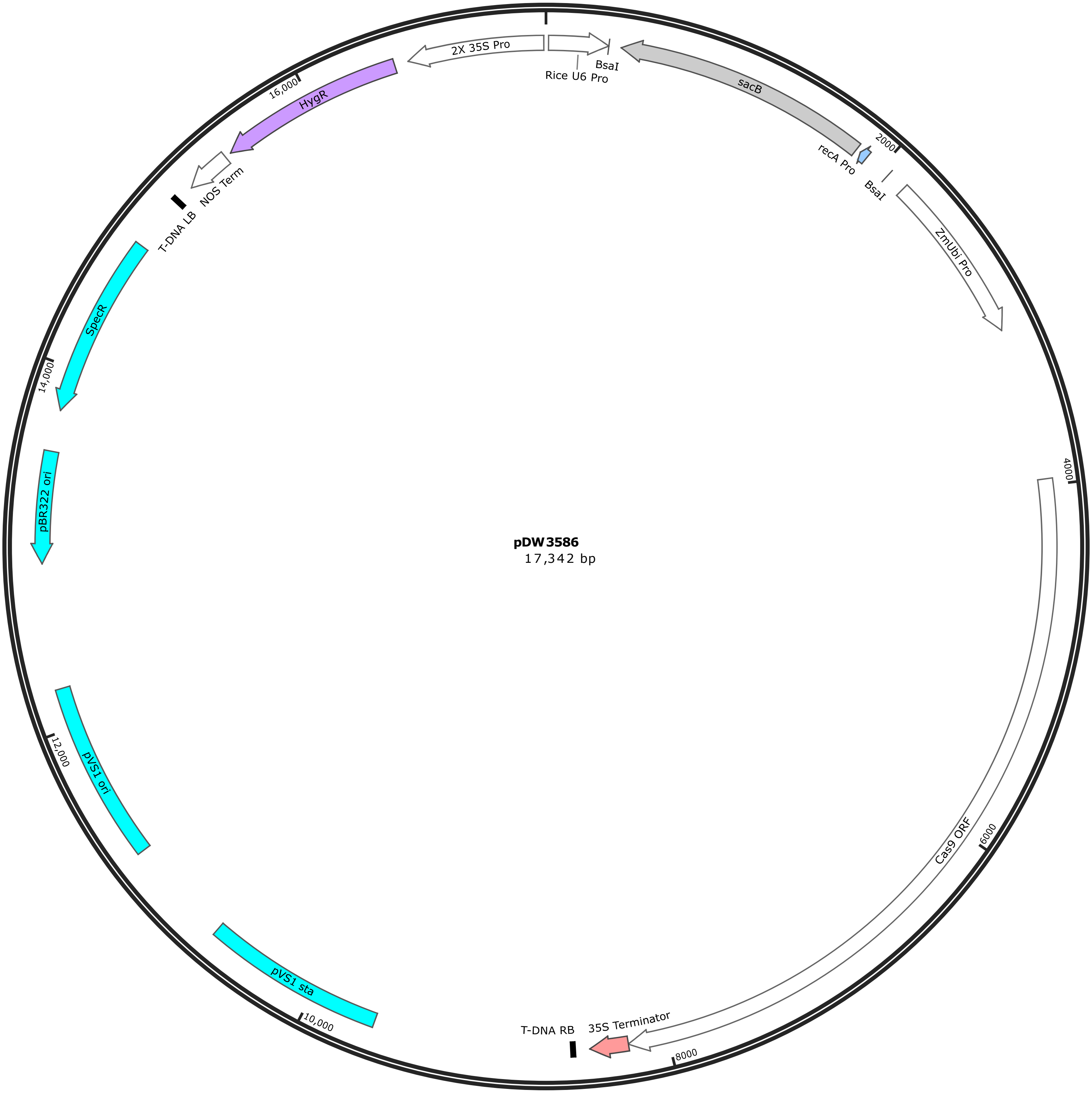
pDW3586
17,342 bp
2X 35S Pro
Rice U6 Pro
BsaI
sacB
recA Pro
BsaI
ZmUbi Pro
Cas9 ORF
35S Terminator
T-DNA RB
pVS1 sta
pVS1 ori
pBR322 ori
SpecR
T-DNA LB
NOS Term
HygR
2000
4000
6000
8000
10,000
12,000
14,000
16,000
